# Deep and periventricular white matter hyperintensities exhibit differential metabolic profiles in arteriosclerotic cerebral small vessel disease: an untargeted metabolomics study

**DOI:** 10.3389/fnins.2025.1607242

**Published:** 2025-05-21

**Authors:** Shisheng Ye, Kaiyan Feng, Guofang Zeng, Jiaxin Cai, Lijuan Liang, Jiaxin Chen, Qishan He, Jianhui Mai, Qiaoling Wu, Chunwan Chen, Haifeng Huang, Li Yuan, Hai Chen, Yizhong Li, Hao Li, Xiong Zhang

**Affiliations:** ^1^Department of Neurology, The First Affiliated Hospital, Jinan University, Guangzhou, China; ^2^Department of Neurology, Maoming People’s Hospital, Maoming, China; ^3^The First School of Clinical Medicine, Southern Medical University, Guangzhou, China; ^4^The First School of Clinical Medicine, Guangdong Medical University, Zhanjiang, China; ^5^Department of Integrated Therapy, Maoming People’s Hospital, Maoming, China; ^6^Department of Radiology, Maoming People’s Hospital, Maoming, China

**Keywords:** deep white matter hyperintensities, periventricular white matter hyperintensities, arteriosclerotic cerebral small vessel disease, untargeted metabolomics, weighted gene correlation network analysis

## Abstract

**Introduction:**

Although white matter hyperintensities (WMH) are radiologically classified as deep WMH (DWMH) and periventricular WMH (PVWMH) based on spatial distribution, the distinct metabolic perturbations driving their pathogenesis remain incompletely characterized.

**Methods:**

This study integrated untargeted metabolomics with MRI phenotyping to delineate metabolic perturbations of WMH in arteriosclerotic cerebral small vessel disease (aCSVD) patients (*n = 30*) versus controls (*n = 29*). Plasma metabolic profiles were analyzed using UPLC-MS. Weighted gene correlation network analysis (WGCNA) evaluated associations between metabolite clusters and clinical traits, including DWMH volume, PVWMH volume and total WMH (TWMH) volume.

**Results:**

We identified 15, 16, and 16 key metabolites meeting both differential expression and WGCNA hub criteria for DWMH, PVWMH, and TWMH, respectively. Pathway Enrichment identified α-linolenic acid and linoleic acid metabolism as common pathway perturbed across both WMH categories. Key metabolites of the pathway, including docosahexaenoic acid (DHA) and stearidonic acid (SDA), demonstrated robust inverse associations with WMH volumes in confounder-adjusted linear regression models. Notably, both WMH categories share common metabolites, particularly polyunsaturated fatty acids (PUFA), while PVWMH-specific metabolites were primarily carnitine derivatives, and DWMH-specific metabolites were prostaglandin E2 and etodolac.

**Conclusion:**

These findings offer new insights into the metabolic mechanisms underlying DWMH and PVWMH in aCSVD. However, the cross-sectional nature of the study does not allow for causal conclusions. Future longitudinal studies are needed to validate the temporal relationships between metabolic perturbations and WMH progression.

## 1 Introduction

Cerebral small vessel disease (CSVD) encompasses a spectrum of clinical, imaging, and pathological syndromes caused by structural and functional alterations in the brain’s small arteries, arterioles, capillaries, and venules (Wardlaw et al., 2019). Among the subtypes of CSVD, arteriosclerotic CSVD (aCSVD) is one of the most prevalent and clinically significant (Pantoni, 2010). A hallmark imaging feature of aCSVD is white matter hyperintensities (WMH), which appear as bilateral and symmetrical hyperintense areas on T2-weighted and fluid-attenuated inversion recovery (FLAIR) MRI sequences. Epidemiological studies indicate that approximately 50% of individuals exhibit WMH by the age of 50 (Wen et al., 2009), with prevalence increasing dramatically to 95% by the age of 90 (de Leeuw et al., 2001). WMH are recognized as a significant marker of poor brain health, strongly associated with elevated risks of stroke, cognitive decline, dementia, gait disturbances, and mortality (Debette et al., 2019; Hu et al., 2021; Herrmann et al., 2008).

The pathophysiological mechanisms underlying WMH remain incompletely understood; however, emerging evidence supports a multifactorial etiology. Key mechanisms include chronic hypoperfusion, inflammatory responses, endothelial dysfunction, and blood-brain barrier (BBB) disruption, as demonstrated in recent studies (Fernando et al., 2006; Lin et al., 2017; Hannawi, 2023). These processes likely interact synergistically, leading to white matter damage and the progression of WMH (Ottavi et al., 2023). WMH are further categorized into deep white matter hyperintensities (DWMH) and periventricular white matter hyperintensities (PVWMH) based on their anatomical locations, each exhibiting distinct functional and histopathological correlates. DWMH are primarily associated with mood disorders and are linked to vascular ischemic injury, characterized by demyelination and myelin rarefaction. In contrast, PVWMH are more frequently associated with cognitive impairment and may arise from non-ischemic mechanisms, such as extracellular fluid accumulation, ependymal disruption, or chronic hemodynamic insufficiency (Huang et al., 2024). These regional differences underscore the involvement of distinct pathological processes in DWMH and PVWMH, which differentially impact brain function and structure. Further research is needed to elucidate the precise molecular pathways and therapeutic targets involved in WMH pathogenesis.

Recent advances in metabolomics have identified a range of circulating metabolites associated with WMH, revealing significant sex-specific differences in these associations (Sliz et al., 2022). For instance, metabolomic profiling has demonstrated that levels of glycerophospholipids and sphingolipids are inversely correlated with WMH volume and cognitive impairment, whereas levels of creatine and sphingosine show positive correlations with WMH burden and cognitive decline (Harshfield et al., 2022). These findings suggest that these metabolites may play a critical role in the pathological processes underlying WMH and their associated cognitive dysfunction, potentially through mechanisms involving energy metabolism dysregulation, oxidative stress, and vascular endothelial dysfunction. Such insights highlight the potential of metabolomics in identifying novel biomarkers and therapeutic targets for WMH.

This study leverages untargeted metabolomics to (1) identify critical metabolic signatures distinguishing aCSVD patients with severe WMH from controls, (2) delineate category-specific (DWMH vs. PVWMH) metabolic dysregulations, and (3) evaluate candidate biomarkers for neurovascular protection. By integrating MRI phenotyping with systems-level metabolomics, we aim to unravel mechanistic insights and therapeutic targets for WMH in aCSVD.

## 2 Materials and methods

This was a cross-sectional study. The study protocol was approved by the Ethics Committee of Maoming People’s Hospital (PJ2020MI-K185-01), and all participants provided written informed consent in accordance with the Helsinki Declaration.

### 2.1 Participants

From October 2022 to December 2024, we recruited 30 patients with severe WMH (Fazekas scores 3) in aCSVD and 30 age- and gender-matched healthy controls from the Department of Neurology at Maoming People’s Hospital.

Inclusion Criteria of WMH in aCSVD: (1) age ≥ 60 years; (2) MRI findings consistent with STRIVE (STandards for ReportIng Vascular changes on nEuroimaging)-defined neuroimaging criteria for cerebral small vessel disease (Duering et al., 2013); (3) presence of ≥1 atherosclerotic risk factor(s): hypertension, diabetes mellitus, hyperlipidemia, current smoking (≥10 cigarettes/day), obesity (BMI > 28 kg/m^2^), hyperhomocysteinemia, or documented Atherosclerotic Cardiovascular Disease (ASCVD); (4) MRI evidence of strictly deep cerebral microbleeds (basal ganglia, thalamus, brainstem, or cerebellar dentate nuclei), excluding lobar or cerebellar cortical microbleeds; (5) severe white matter hyperintensities (WMH) on FLAIR imaging, classified as Fazekas grades 3. Exclusion Criteria: (1) Alternative cerebral small vessel disease etiologies: Cerebral amyloid angiopathy (sporadic or hereditary); Monogenic small vessel diseases; Inflammatory/immune-mediated vasculopathies; (2) Hemodynamically significant intracranial atherosclerotic stenosis (>50% luminal narrowing in major cerebral arteries); (3) Ischemic stroke attributable to large artery atherosclerosis or cardioembolic sources; (4) Secondary neurological pathologies (infectious, metabolic, toxic, neoplastic, or post-traumatic etiologies); (5) Acute/chronic intracerebral hemorrhage (parenchymal hematoma volume > 10 mL); (6) Major systemic comorbidities: Active pulmonary infection; Decompensated heart failure (NYHA class ≥ II); Severe renal impairment (eGFR < 30 mL/min/1.73 m^2^); Hepatocellular dysfunction (ALT/AST > 3 × ULN or total bilirubin > 3 × ULN).

### 2.2 MRI parameters and analysis pipeline

All participants underwent neuroimaging on a 3.0-T Discovery MR750 scanner (General Electric, Milwaukee, USA) equipped with an 8-channel HRBRAIN head coil. The standardized protocol included: Axial T1 FLAIR-weighted imaging (TR/TE = 1,750/24 ms, echo train length ETLETL = 10, bandwidth BWBW = 41.67 kHz, matrix = 320 × 224, FOV = 240 × 240 mm^2^, slice thickness/gap = 5/1 mm, NEX = 1); Axial T2 PROPELLER (FrFSE) (TR/TE = 5,727/93 ms, ETL = 32, BW = 83.3 kHz, matrix = 512 × 512, FOV = 240 × 240 mm^2^, slice thickness/gap = 5/1 mm, NEX = 1.5); T2 FLAIR (TR/TE/TI = 8,400/145/2,100 ms, BW = 83.3 kHz, flip angle = 145°, matrix = 320 × 224, FOV = 240 × 240 mm^2^, slice thickness/gap = 5/1 mm, NEX = 1); 3D Time-of-Flight MR Angiography (TOF-MRA) (TR/TE = 25/3.4 ms, flip angle = 20°, BW = 41.67 kHz, matrix = 384 × 320, FOV = 200 × 200 mm^2^, isotropic voxel size = 0.8 mm^3^, NEX = 1); Axial T2-weighted SWAN* (TR/TE = 77.3/45 ms, BW = 62.5 kHz, flip angle = 15°, matrix = 384 × 320, slice thickness = 2 mm, NEX = 1).

**FIGURE 1:**
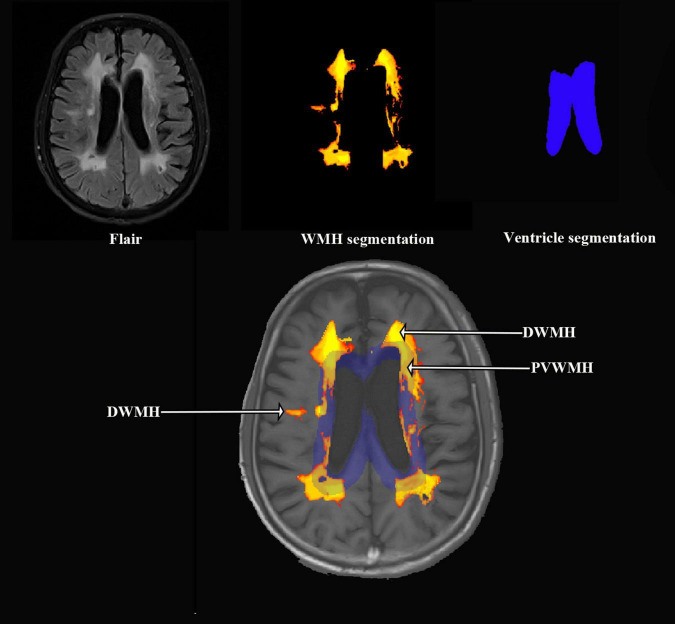
Representation of the image processing pipeline.

The MRI analysis pipeline was designed to quantify WMH. The processing steps were as follows: (1) Image Registration: The FLAIR and T1-weighted images were co-registered using the LST (Lesion Segmentation Tool, version 3.0.0) toolbox^1^ within SPM12 (Statistical Parametric Mapping, version 12) implemented in MATLAB (version 9.9). This step ensured spatial alignment between the two imaging modalities, which is essential for accurate segmentation and subsequent analysis. (2) Total WMH Volume Segmentation: Following registration, the total WMH volume was automatically segmented from the co-registered FLAIR images by the lesion prediction algorithm (Schmidt, 2017). (3) Lateral Ventricle and PVWMH Segmentation:The lateral ventricles and PVWMH were segmented using the anatomical tools from the FSL (FMRIB Software Library, version 6.0) (Kempton et al., 2011). The lateral ventricle masks were generated. PVWMH were defined as lesions less than 10 mm distance from the ventricles, otherwise it is DWMH (Ludovica et al., 2017). (4) DWMH Volume Calculation: The volume of DWMH was calculated by subtracting the PVWMH volume from the total WMH volume. (5) Quality Control: Visual quality checks were performed on both the original and processed MRI images to ensure accuracy. Any cases with oversegmentation, artifacts, or incorrect segmentations were excluded from further analysis. Figure 1 offers a diagrammatic representation of the image processing pipeline.

### 2.3 Serum sample collection and sample preparation

Overnight fasting venous blood specimens were collected from all subjects. The samples were placed in serum separation tubes and centrifuged at 1500 rpm for 10 min at 4°C. The resulting serum supernatant was aseptically aliquoted into pre-labeled cryogenic vials and stored at −80°C in ultra-low temperature freezers until subsequent biochemical analysis.

Serum samples were thawed at 4°C until completely liquefied. For metabolite extraction, 100 μL aliquots of each sample, including quality control (QC) samples prepared by pooling equal volumes from all specimens, were transferred to 1.5 mL Eppendorf tubes. Then, 700 μL of ice-cold extraction solvent (methanol:acetonitrile:water = 4:2:1,v/v/v) was added. The mixture was vortexed vigorously for 1 min and incubated at −20°C for 2 h to precipitate proteins. After centrifugation at 25000 rpm for 15 min at 4°C, 600 μL of the supernatant was transferred to fresh tubes. The supernatant was lyophilized using a vacuum concentrator and reconstituted in 180 μL of methanol:water (1:1, v/v). After vortex mixing for 10 min and centrifugation under the same conditions (25000 rpm, 4°C, 15 min), the final supernatant was transferred to LC-MS vials for analysis. QC samples were generated by combining 20 μL aliquots from each prepared sample to monitor system stability and reproducibility throughout the analytical sequence.

### 2.4 Metabolomics measurement

Chromatographic separation was performed using a Vanquish UPLC system (Thermo Scientific) coupled to an Orbitrap Exploris 480 mass spectrometer operated in dual polarity mode. Mass spectra were acquired in full scan mode (m/z 70–1050) with 120000 resolution (MS1) and 30,500 resolution (MS2), employing stepped collision energy (20/40/60 eV) for data-dependent MS/MS acquisition. Ion source parameters were optimized as follows: sheath gas 40 arb, auxiliary gas 10 arb, spray voltage ± 3.80/3.20 kV (positive/negative mode), capillary temperature 320°C, and auxiliary heater 350°C. Raw data were processed through Compound Discoverer 3.3 (Thermo Scientific) using multi-database matching (BMDB (Beijing Genomics institution metabolome database), mzCloud^2^, ChemSpider^3^ with strict mass tolerance thresholds (<5 ppm precursor, <10 ppm fragment) and retention time alignment (<0.2 min deviation).

The offline mass spectrometry data were imported into Compound Discoverer 3.3 (Thermo Fisher Scientific, USA) and analyzed in conjunction with the BMDB database, mzCloud database, and ChemSpider online database. This process yielded a data matrix containing metabolite peak area and identification results.

### 2.5 Data process and metabolomics analysis

The results from Compound Discoverer were input into MetaX for data preprocessing and further analysis. The preprocessing steps included: (1) Normalizing the data using Probabilistic Quotient Normalization (PQN) to obtain relative peak areas. (2) Correcting batch effects using Quality control-based robust LOESS signal correction. (3) Removing metabolites with a Coefficient of Variation exceeding 30% in their relative peak area within QC samples.

To detect group differences, unsupervised principal component analysis (PCA) and supervised orthogonal partial least-squares-discriminant analysis (OPLS-DA) were employed. All the models evaluated were tested for overfitting with methods of permutation tests (*n* = 200). Variable influence on projection (VIP) values of metabolites were obtained. Student’s *t*-test was used to assess the significance of metabolite expression differences in each comparison group, yielding *p*-values. These *p*-values were corrected using the Benjamini-Hochberg algorithm to obtain false discovery rate (FDR) adjusted *p*-value. Metabolites with VIP > 1.0, fold change (FC, WMH/controls) > 1.2 or <0.83, and FDR adjusted *p*-value < 0.05 were considered as differential metabolites. Metabolites were annotated using the Kyoto Encyclopedia of Genes and Genomes (KEGG)^4^ and Human Metabolome Database (HMDB)^5^, providing information such as KEGG ID, HMDB ID, category, and involvement in KEGG metabolic pathways.

### 2.6 Weighted gene correlation network analysis

Weighted gene correlation network analysis (WGCNA), a systems biology method, has been applied in many high-dimensional data sets, including metabolomics (Jiang et al., 2025). In the current study, WGCNA was implemented to investigate metabolome-wide associations with WMH categories (DWMH, PVWMH and total WMH). This methodology involves four key analytical phases (Langfelder and Horvath, 2008): (1) Construction of a signed metabolite co-expression network using soft-thresholding power to preserve biological meaningfulness while minimizing spurious connections; (2) Hierarchical clustering of metabolites through topological overlap matrix (TOM)-based dissimilarity measures to identify cohesive metabolite modules; (3) Module-trait association analysis to identify biologically relevant modules showing significant correlations (*p* < 0.05) with WMH categories; and (4) Identification of intramodular hub metabolites through dual topological criteria.

Hub metabolites were operationally defined as those demonstrating both high intramodular connectivity (module membership [MM] > |0.8|) and significant phenotypic association (gene significance [GS] > |0.2|). These stringent thresholds ensure selection of metabolites that not only occupy central positions in the interaction network but also exhibit strong biological relevance to WMH pathophysiology. The MM metric quantifies intramodular connectivity through Pearson correlation between metabolite expression profiles and module eigengenes, while GS represents the absolute correlation between individual metabolite levels and clinical traits of interest.

### 2.7 Statistical analysis

Statistical analyses were conducted using metaX software (Wen et al., 2017) and MATLAB R2023b. Linear regression models were constructed to assess relationships between key metabolites and WMH categories, incorporating adjustment for age, sex, hypertension, diabetes, hyperlipoidemia and BMI. The normality assumption was evaluated based on the residuals and confirmed visually and calculated using the Shapiro-Wilk test. If the normality assumption was not fulfilled the WMH volumes were log transformed, after which normality assumption was met. Statistical significance was defined as *P* < 0.05 for clinical variables and Benjamini-Hochberg false discovery rate (FDR)-adjusted *P* < 0.05 for metabolomics data, ensuring robust control for multiple comparisons.

## 3 Results

### 3.1 Baseline characteristics of study population

The study comprised 30 patients with severe WMH and 29 age- and gender-matched healthy controls, with one control excluded due to hemolytic serum interference (Table 1). Demographic profiles, including age, sex distribution, and vascular risk factors (hypertension, diabetes, hyperlipidemia, BMI), were comparable between groups. Volumetric analysis revealed significantly larger WMH volumes in the WMH group compared to controls across all categories.

**TABLE 1:** Demographics of the participants and distribution of WMH volume.

Number of subjects	WMH (*n* = 30)	HC (*n* = 29)	*p*
Age (y)	69.2 ± 6.4	67.5 ± 5.6	0.276
Male, *n* (%)	19 (63.3)	18 (62.1)	0.920
Hypertension, *n* (%)	28 (93.3)	0 (0)	<0.001
Diabetes mellitus, *n* (%)	6 (20.0)	0 (0)	0.024
Hyperlipidemia, *n* (%)	12 (40.0)	15 (51.72%)	0.366
Body mass index (kg/m^2^)	22.69 (21.43, 23.85)	23.14 (20.25, 24.55)	0.872
DWMH volume (ml)	5.41 (4.14, 9.44)	0.12 (0.01, 0.32)	<0.001
PVWMH volume (ml)	19.77 (15.84, 31.11)	1.16 (0.49, 2.97)	<0.001
TWMH volume (ml)	27.58 (20.11, 40.92)	1.41 (0.52, 3.74)	<0.001

DWMH, deep white matter hyperintensities; PVWMH, periventricular white matter hyperintensities; TWMH, total white matter hyperintensities.

### 3.2 Quality control of UPLC-MS analytical performance

Total ion chromatograms (TIC) demonstrated high reproducibility in retention time (RT) alignment and peak area consistency across quality control (QC) samples in both positive (Supplementary Figure 1A) and negative (Supplementary Figure 1B) ionization modes. The coefficient of variation (CV) for QC metabolites was <15%, confirming robust system stability and data reliability.

### 3.3 Plasma metabolomics multivariate statistical analysis

**FIGURE 2:**
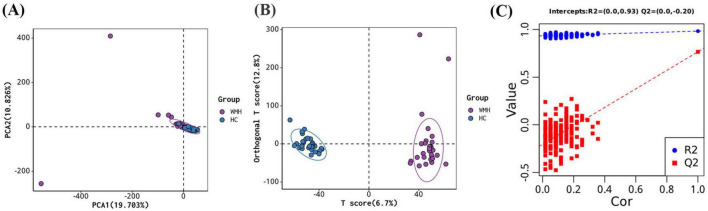
Multivariate analysis for metabolites. **(A)** Principal component analysis (PCA) score plots; **(B)** orthogonal projections to latent structures—discriminant analysis (OPLS-DA) score plots; **(C)** permutation test of OPLS-DA.

In total, 1978 metabolites were identified based on LC-MS/MS spectra. An unsupervised PCA analysis demonstrated clear clustering of the WMH patients and controls (Figure 2A). However, a supervised OPLS-DA model offered superior discrimination of metabolic profiles between the WMH patients and controls (Figure 2B). Furthermore, seven rounds of cross - validation and 200 rounds of RPT confirmed the robustness of the OPLS-DA models, with the R2Y and Q2 values of 0.983 and 0.769, respectively (Figure 2C).

### 3.4 Differential metabolite screening

**FIGURE 3:**
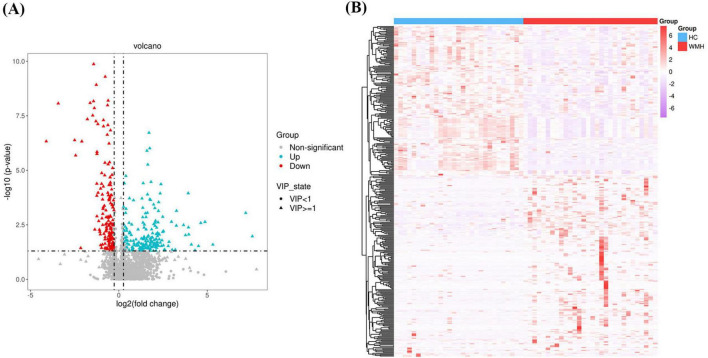
Volcano plots **(A)** and heatmaps **(B)** illustrating the different metabolites.

A multistep analytical framework was implemented to identify differentially expressed metabolites. First, univariate analysis combining OPLS-DA and Student’s *t*-test identified 359 preliminary differential metabolites (FDR-adjusted *p* < 0.05, VIP > 1). Volcano plot visualization (Figure 3A) refined this set using dual thresholds: (1) statistical significance (FDR < 0.05), and (2) biological relevance (|log2 fold change| > 0.26, equivalent to ±20% expression variation). This stratified 353 candidate metabolites, comprising 185 upregulated and 168 downregulated metabolites in WMH patients versus controls (Supplementary Table 1). Expression-derived metrics (EDM), calculated as Z-score normalized abundance values, were subjected to unsupervised hierarchical clustering (complete linkage method, Euclidean distance) to reveal co-regulation patterns (Figure 3B).

### 3.5 WGCNA

**FIGURE 4:**
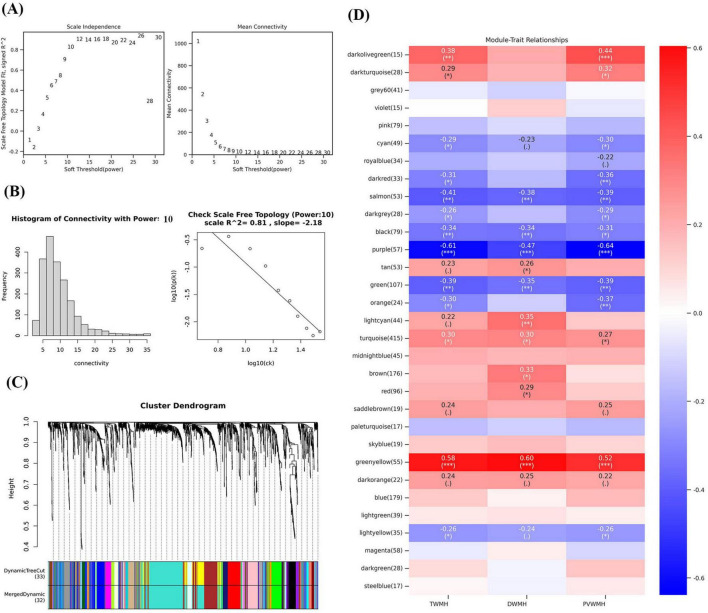
Weighted correlation network analysis and the selection of hub metabolites. **(A)** Scale-free fitting index analysis and mean connectivity of soft threshold power from 1 to 30. **(B)** Checking the scale free topology (power = 10). **(C)** Clustering dendrograms. **(D)** Correlation heatmap between module eigengenes and clinical traits. *P < 0.05; **P < 0.01; ***P < 0.001.

WGCNA was performed using a soft-thresholding power of 10, selected as the minimum value achieving scale-free topology (scale-free fit index ≥ 0.80; Figures 4A, B). Hierarchical clustering with dynamic tree cutting identified 32 co-expression modules (Figure 4C), where the gray module represented unclassified metabolites. Module-trait association analysis revealed distinct correlation patterns across WMH categories (Figure 4D). DWMH showed significant negative correlations with salmon (*r* = −0.38, *P* = 0.0031), black (*r* = −0.34, *P* = 0.0076), purple (*r* = −0.47, *P* = 1.73 × 10^–4^) and green (*r* = −0.35, *P* = 0.0070) modules, while exhibiting positive correlations with lightcyan (*r* = 0.35, *P* = 0.0060) and greenyellow (*r* = 0.60, *P* = 4.05 × 10^–7^) modules. Similarly, PVWMH demonstrated negative correlations with salmon (*r* = −0.39, *P* = 0.0021), darkred (*r* = −0.36, *P* = 0.0053), purple (*r* = −0.64, *P* = 5.23 × 10^–8^), orange (*r* = −0.37, *P* = 0.0044) and green (*r* = −0.39, *P* = 0.0025) modules, along with positive correlations for darkolivegreen (*r* = 0.44, *P* = 5.60 × 10^–4^) and greenyellow (*r* = 0.52, *P* = 2.57 × 10^–5^) modules. TWMH exhibited comparable patterns, with strong negative correlations to salmon (*r* = −0.41, *P* = 0.0013), black (*r* = −0.34, *P* = 0.0080), purple (*r* = −0.61, *P* = 2.50 × 10^–7^) and green (*r* = −0.39, *P* = 0.0020) modules, and positive correlations with darkolivegreen (*r* = 0.38, *P* = 0.0031) and greenyellow (*r* = 0.59, *P* = 1.58 × 10^–6^) modules. These significantly associated modules were prioritized for downstream analysis, with intramodular hub metabolites identified using dual thresholds (module membership [MM] ≥ 0.80 and |gene significance [GS]| ≥ 0.20). The MM-GS relationships for these metabolites were visualized in scatter plots (Supplementary Figure 2), confirming their central roles in network topology and phenotypic associations.

Through rigorous network topology screening, we identified 86, 77, and 94 hub metabolites significantly associated with DWMH, PVWMH and TWMH volumes, respectively. Notably, only 29, 32, and 32 of these metabolites were annotated in the HMDB (Supplementary Table 2), while the purple and lightcyan modules contained no identifiable metabolites—a finding suggestive of novel biochemical pathways in these network clusters. Strikingly, the green modules across all WMH categories exhibited pronounced enrichment of polyunsaturated fatty acids (PUFA), including ω-3 (stearidonic acid (SDA) [18:4n-3]) and ω-6 derivatives (5E,8E,11E-hexadecatrienoic acid [16:3n-1], linoleic acid (LA) [18:2n-6], γ-linolenic acid [18:3n-6], dihomo-α-linolenic acid [20:3n-6], and adrenic acid [22:4n-6]). These modules also contained ω-7 monounsaturated (palmitoleic acid [16:1n-7]) and saturated fatty acids (myristic acid [14:0]), oxylipins (8(S)-hydroxy-eicosatetraenoic acid [8(S)-HETE]), hydroxylated lipids (3-hydroxy myristic acid [3-OH-MA], 9-hydroxyoctadecanoic acid [9-HOA]), and the steroid androsterone.

Category-specific metabolic signatures revealed divergent pathobiological mechanisms: PVWMH-associated metabolites clustered in orange/darkolivegreen/darkred modules were dominated by carnitine derivatives (hexanoylcarnitine [C6], trans-2-dodecenoylcarnitine [C12:1], myristoleoylcarnitine [C14:1], 9-hexadecenoylcarnitine [C16:1], palmitoylcarnitine [C16]) alongside mevalonic acid, ursolic acid, and finasteride. In contrast, DWMH-specific metabolites within the black module comprised prostaglandin E2 (PGE2), etodolac, loperamide, flurandrenolide, minoxidil, and alfentanil.

### 3.6 Key metabolites identification and pathway enrichment

**FIGURE 5:**
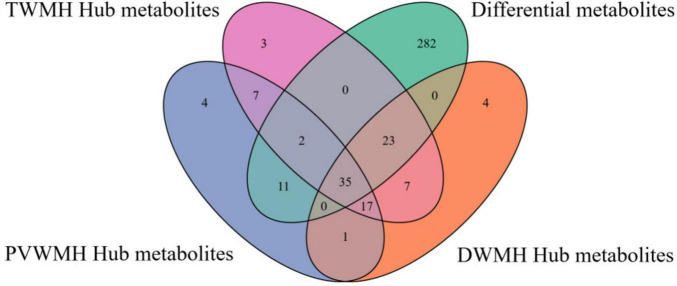
The Venn diagram of differential and hub metabolites.

**FIGURE 6:**
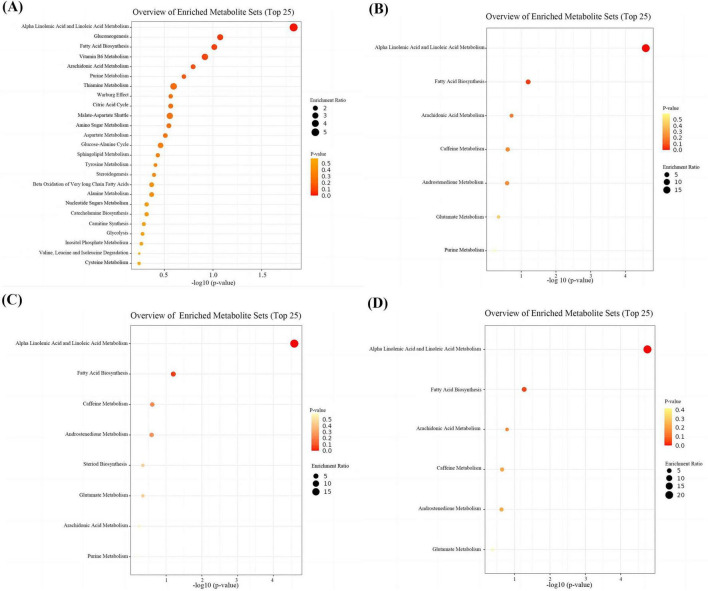
Enrichment analyses of differential and key metabolites. **(A)** Enrichment analyses of differential metabolites. **(B)** Enrichment analyses of key metabolites for DWMH. **(C)** Enrichment analyses of key metabolites for PVWMH. **(D)** Enrichment analyses of key metabolites for TWMH.

Through integrative analysis of differential expression (|log2 fold change| > 0.26, FDR < 0.05, VIP > 1) and network centrality ([MM] ≥ 0.80 and |[GS]| ≥ 0.20), we identified 15, 16, and 16 key metabolites for DWMH, PVWMH, and TWMH respectively, including nine conserved across all categories: ω-3/6 polyunsaturated fatty acids (SDA, 5E,8E,11E-hexadecatrienoic acid, γ-linolenic acid), myristic acid, hydroxylated lipids [(R)-3-hydroxy myristic acid, 8(S)-HETE], and secondary metabolites (purine, 3-hydroxy-3-methylglutaric acid, catechin) (Figure 5 and Supplementary Tables 1, 2). Pathway enrichment analysis revealed α-linolenic acid and linoleic acid metabolism as the core perturbed pathways, with coordinated downregulation of five critical intermediates—LA, γ-linolenic acid, SDA, adrenic acid, and docosahexaenoic acid (DHA) (22:6n-3) (Figures 6A–D and Supplementary Table 3).

### 3.7 Linear regression models for WMH volume

Linear regression models adjusted for age, sex, hypertension, diabetes, hyperlipidemia, and BMI were employed to evaluate associations between five key metabolites and WMH volume (Table 2). DHA exhibited significant inverse associations with all WMH categories: DWMH: β = −0.575, 95% CI [−0.937, −0.214], R^2^ = 0.30, *P* = 0.003), PVWMH: β = −0.302, 95% CI [−0.550, −0.054], R^2^ = 0.03, *P* = 0.019), and TWMH: β = −0.363, 95% CI [−0.614, −0.112], R^2^ = 0.15, *P* = 0.007), indicating its broad protective role against lesion progression. SDA was inversely associated with DWMH (β = −0.565, 95% CI [−0.895, −0.235], R^2^ = 0.33, *P* = 0.002) and TWMH volumes (β = −0.268, 95% CI [−0.520, −0.016], R^2^ = 0.01, *P* = 0.038), while LA showed a negative association specifically with DWMH (β = −0.451, 95% CI [−0.890, −0.012], R^2^ = 0.13, *P* = 0.044).

**TABLE 2:** Linear regression models for WMH volume.

Metabolites	Log DWMH			Log PVWMH			Log TWMH		
	β (95% CI)	R^2^	*P*	β (95% CI)	R^2^	*P*	β (95% CI)	R^2^	*P*
Stearidonic acid	−0.565 (−0.895, −0.235)	0.33	0.0017	−0.203 (−0.451, 0.045)	−0.10	0.10	−0.268 (−0.520, −0.016)	0.01	0.038
Adrenic acid	−0.085 (−0.479, 0.308)	−0.04	0.65	−0.020 (−0.272, 0.232)	−0.24	0.86	−0.049 (−0.315, 0.216)	−0.19	0.70
γ-Linolenic acid	−0.370 (−0.743, 0.003)	0.12	0.051	−0.163 (−0.413, 0.087)	−0.15	0.18	−0.209 (−0.468, 0.050)	−0.06	0.10
Linoleic acid	−0.451 (−0.890, −0.012)	0.13	0.044	−0.156 (−0.456, 0.143)	−0.18	0.29	−0.231 (−0.540, 0.077)	−0.08	0.13
Docosahexaenoic acid	−0.575 (−0.937, −0.214)	0.30	0.0032	−0.302 (−0.550, −0.054)	0.03	0.019	−0.363 (−0.614, −0.112)	0.15	0.0066

DWMH, deep white matter hyperintensities; PVWMH, periventricular white matter hyperintensities; TWMH, total white matter hyperintensities.

## 4 Discussion

This study integrates untargeted metabolomics with MRI phenotyping to elucidate critical metabolic perturbations underlying WMH in aCSVD. Through a multi-tiered analytical framework—combining differential metabolite screening, WGCNA, and confounder-adjusted linear regression models—we identified α-linolenic acid and linoleic acid metabolism as core dysregulated pathways across all WMH categories. These pathways, central to PUFA homeostasis, exhibited profound alterations that may mechanistically drive WMH pathogenesis. Notably, systemic depletion of neuroprotective PUFA, including DHA and SDA, demonstrated robust inverse associations with WMH volumes in linear regression models, independent of age, sex, and vascular risk factors. These findings implicate PUFA dysregulation as a pivotal driver of neurovascular injury while unveiling metabolic signatures with therapeutic potential. By bridging metabolomic perturbations to imaging phenotypes, this work advances mechanistic insights into WMH and establishes a foundation for targeted biomarker discovery and lipid-centric therapeutic strategies.

The interplay between PUFA and WMH centers on oxidative stress, neuroinflammation, and vascular dysfunction. Oxidative stress is driven by soluble epoxide hydrolase (sEH)-mediated conversion of anti-inflammatory ω-6 PUFA epoxides (EpOME) into pro-oxidative metabolites (e.g., 12,13-DiHOME), with elevated sEH activity exacerbating vascular damage and correlating with WMH severity (Yu et al., 2019; Yu et al., 2023; Shinto et al., 2020). Clinical data link increased 12,13-DiHOME/EpOME ratios and lipid peroxidation markers (e.g., LPH) to microvascular injury in CSVD (Yu et al., 2023; Swardfager et al., 2017), while sEH inhibition mitigates oxidative stress and improves endothelial function (Yu et al., 2019; Shinto et al., 2020). Neuroinflammation involves ω-3 PUFA suppressing NF-κB-dependent cytokines (IL-6, TNF-α), countering ω-6-derived pro-inflammatory mediators. sEH further amplifies inflammation by degrading anti-inflammatory EETs, with its activity positively associated with IL-1β in WMH patients (Yu et al., 2019; Shinto et al., 2020; McNamara and Almeida, 2019). Animal studies demonstrate reduced WMH pathology in sEH knockout mice and therapeutic benefits of ω-3 supplementation (Yu et al., 2019; Bowman et al., 2019; Rouch et al., 2022). Vascular dysfunction arises from oxidative impairment of nitric oxide (NO)-mediated vasodilation, inversely correlating with WMH severity (Shibata et al., 2004), while DHA enhances blood-brain barrier integrity via occludin upregulation, contrasting with ω-3 deficiency-induced leakage and demyelination (Tan et al., 2012; McNamara and Almeida, 2019). Future research should prioritize lipidomic profiling of sEH metabolites, gene-edited models, and co-culture systems to refine targeted interventions.

DHA, a long-chain ω-3 PUFA, emerged as a biomarker with neuroprotective associations across all WMH categories. PUFA, particularly ω-3 and ω-6 subtypes, are diet-derived lipids structurally integrated into brain cell membranes and myelin, critically regulating synaptic signaling, neuroinflammation modulation, and cerebral glucose metabolism (Tian et al., 2025). Observational studies consistently associate higher plasma ω-3 PUFA levels with reduced WMH burden and preserved cognitive performance, particularly in executive function (Loong et al., 2023; Yu et al., 2019). A large-scale cohort study further linked elevated PUFA intake to decreased dementia risk and WMH progression (He et al., 2023). However, interventional trials yield conflicting results: Shinto et al. (2024) observed benefits limited to APOEε4 carriers. These discrepancies underscore the need for precision trials incorporating genetic stratification (e.g., APOE genotype) and optimized dosing regimens to clarify therapeutic efficacy.

Our study revealed a significant inverse correlation between SDA and DWMH/TWMH volumes. As a metabolic intermediate of α-linolenic acid, SDA enhances eicosapentaenoic acid (EPA) incorporation into membrane phospholipids, exerting anti-inflammatory and antioxidant effects (Harris, 2012; Prasad et al., 2021). While direct evidence linking stearidonic acid to WMH remains sparse, its role in mitigating neuroinflammation and oxidative stress aligns with its observed protective associations. Future studies should delineate SDA’s mechanistic contributions to CSVD pathology and evaluate its therapeutic potential.

In parallel, LA, an essential ω-6 PUFA, exhibited a marginal negative association with DWMH. Despite its physiological necessity, excessive LA intake may promote neuroinflammation through oxidized metabolites (e.g.,OXLAMs) (Taha, 2020). Paradoxically, short-term studies suggest LA transiently suppresses microglial inflammation (Tu et al., 2019), while its derivatives (e.g., dihydroxyoctadecenoic acids) disrupt endothelial function in CSVD (Yu et al., 2023). Clinically, LA-enriched lipids correlate with reduced ischemic stroke risk but elevated intracerebral hemorrhage incidence (Zhang et al., 2020; Zhang et al., 2024). These divergent effects highlight the importance of maintaining an optimal ω-3/ω-6 balance, warranting further investigation into LA’s metabolic interplay with ω-3 PUFA.

The therapeutic potential of PUFA supplementation for WMH requires rigorous clinical evaluation. Clinical trial outcomes indicate that efficacy can vary based on dosage, duration, and patient stratification. For example, Shinto et al. (2024) found that while 1.65 g/d of ω-3 PUFA for 3 years didn’t significantly reduce WMH progression in all participants, it did reduce neuronal integrity breakdown in APOE*E4 carriers. When evaluating PUFA supplementation’s risk-benefit profile, potential pro-oxidant effects at high doses and drug interactions should be considered. High doses ω-6 PUFA can worsen white matter damage via lipid peroxidation products (Shinto et al., 2020; Swardfager et al., 2017), and ω-3/ω-6 metabolic derivatives (e.g., oxylipins) have pro-inflammatory and anti-inflammatory properties (Yu et al., 2023). Moreover, PUFA may increase bleeding risk from anticoagulants (Okumura et al., 2024; Bowman et al., 2019) and interfere with antihypertensive drug efficacy by altering sEH enzyme activity (Yu et al., 2023; Shinto et al., 2020). Regulatory considerations for PUFA dietary supplements are crucial, as commercially available supplements exhibit wide variability in EPA/DHA ratios. Independent batch testing [as performed in Shinto et al. (2024)] should be mandated to ensure potency and purity.

These insights converge with established mechanisms driving WMH, integrating neuroinflammation, endothelial dysfunction, chronic hypoperfusion, and BBB disruption into a cohesive pathological framework. First, neuroinflammation-characterized by microglial activation and cytokine release—is mitigated by ω-3 PUFA through dual pathways: (1) GPR120-mediated suppression of NF-κB nuclear translocation, blocking pro-inflammatory signaling (Chang et al., 2021; Nakajima et al., 2023), and (2) PPAR pathway activation by DHA and its metabolite neuroprotectin D1 (NPD1), reducing oxidative stress and neuronal damage (Bosviel et al., 2017). Concurrently, endothelial dysfunction, a hallmark of CSVD, is counteracted by DHA through enhanced NO bioavailability via AMPK activation, attenuated expression of adhesion molecules (ICAM-1/VCAM-1), and reduced endothelial lipotoxicity from triglyceride-rich lipoproteins (Yamada et al., 2008; Wu et al., 2012; Arabi et al., 2024). Chronic hypoperfusion, particularly in vulnerable watershed zones, is ameliorated by long-chain ω-3 PUFA (e.g., EPA and DHA), which improve cerebral perfusion via endothelial-dependent vasodilation while suppressing oxidative stress and platelet aggregation (Kuszewski et al., 2017; Schwarz et al., 2018). Finally, BBB integrity—critical for neurovascular homeostasis—is reinforced by DHA through upregulation of tight junction proteins (e.g., ZO-1) and reduced neuroinflammation (Xie et al., 2020; Wen et al., 2024). These interconnected mechanisms collectively highlight the central role of PUFA dysregulation in WMH pathogenesis, bridging molecular pathways to imaging phenotypes.

Crucially, our metabolomic profiling identified distinct category-specific metabolic signatures: PVWMH was characterized by carnitine derivatives (e.g., palmitoylcarnitine and hexanoylcarnitine), while DWMH exhibited unique associations with prostaglandin E2 (PGE2) and etodolac. The divergent metabolic signatures associated with DWMH and PWMH highlight their distinct pathogenesis. Carnitine derivatives may indirectly influence non-ischemic cerebrospinal fluid (CSF) accumulation and ependymal function by modulating mitochondrial energy metabolism, reducing oxidative damage, and suppressing inflammatory responses. Abnormal carnitine profiles in hydrocephalus patients suggest that metabolic imbalance may be one of the contributing factors to fluid accumulation (Temiz et al., 2025). Furthermore, the energy-dependent nature of ependymal cells and their susceptibility to lipotoxicity render them vulnerable to disruptions in carnitine metabolism (Manzo et al., 2018). In contrast, DWMH is linked to COX-2-derived PGE2, which induces microvascular endothelial dysfunction via EP receptor activation, while etodolac—by inhibiting COX-2—ameliorates microcirculatory impairment (Zhang et al., 2025; Liu et al., 2020). Therefore, we hypothesize that the shared pathological mechanism between DWMH and PVWMH lies in PUFA metabolic dysregulation, while DWMH-specific pathology is driven by ischemic damage and microvascular pathology, and PVWMH-specific pathology involves non-ischemic fluid accumulation and ependymal disruption.

The associations between WMH and perturbed α-linolenic/linoleic acid metabolism can be explained by two interconnected mechanistic frameworks: gut-brain axis dysregulation and mitochondrial dysfunction. These pathways form the basis of our hypothesis that systemic metabolic disturbances drive neurovascular injury and WMH progression through inflammatory, oxidative, and bioenergetic mechanisms. Previous research has shown that the composition and function of the gut microbiota are closely related to the metabolism of Omega-3 and Omega-6 fatty acids (Zinkow et al., 2024). The intake and metabolism of α-linolenic acid and linoleic acid can influence the composition of the gut microbiota, which in turn can regulate the host’s metabolism and immune function. Dysbiosis of the gut microbiota may affect brain inflammation and vascular function via the gut-brain axis, thereby contributing to the pathogenesis of WMH. Furthermore, PVWMH-specific carnitine derivatives reflect impaired mitochondrial fatty acid β-oxidation (Manzo et al., 2018). This dysfunction can lead to abnormal cellular energy metabolism and trigger inflammatory responses, which are detrimental to oligodendrocytes and other energy-demanding cells in the white matter, ultimately causing WMH. Additionally, the reactive oxygen species generated by mitochondrial dysfunction can exacerbate cellular damage, worsening WMH (Mahapatra et al., 2023).

## 5 Limitations and future directions

Our study has several limitations. First, the cross-sectional nature of the study prevents us from establishing a causal relationship between metabolic changes and WMH progression. Reverse causation, such as dietary modifications caused by WMH, cannot be ruled out. Future research should include longitudinal cohorts with repeated MRI and metabolomic assessments to clarify the temporal relationships between these factors. Second, the relatively small sample size might compromise the statistical power for subgroup analyses. Notably, the purple and lightcyan modules contained 17 unannotated hub metabolites. These unknown metabolites could be new biochemical entities or intermediate products in pathways related to WMH development. Targeted metabolomic methods focusing on lipid peroxidation products and epoxide derivatives could help identify these metabolites, especially considering the observed enrichment of PUFA-related pathways. Third, while we adjusted for major vascular risk factors, there may be unmeasured confounders that could affect systemic PUFA concentrations and WMH progression. For example, dietary PUFA intake, ω-3/ω-6 ratios, and physical activity levels could all play a role. Fish consumption patterns might directly influence DHA levels, regardless of disease status. Future studies should include detailed dietary assessments and track PUFA levels over time to better understand these relationships.

## 6 Conclusion

By integrating untargeted metabolomics with MRI phenotyping, this study delineates distinct and shared metabolic landscapes underlying DWMH and PVWMH in aCSVD. To confirm causality, it is vital to conduct prospective validation in independent cohorts with repeated MRI and metabolomic analyses. Additionally, incorporating dietary information and targeted lipidomics will be crucial for verifying the role of PUFA homeostasis in WMH pathobiology.

## Data Availability

The raw data supporting the conclusions of this article will be made available by the authors, without undue reservation.

## Footnotes

1
www.statistical-modeling.de/lst.html

2
https://www.mzcloud.org/

3
https://www.chemspider.com/

4
https://www.kegg.jp

5
https://hmdb.ca/metabolites
