# Supplementary material for: Deep and periventricular white matter hyperintensities exhibit differential metabolic profiles in arteriosclerotic cerebral small vessel disease: an untargeted metabolomics study
Source: Front Neurosci. 2025 May 21;19:1607242. doi: 10.3389/fnins.2025.1607242 (PMC12133732; doi:10.3389/fnins.2025.1607242)
Supplement: Supplementary file 1 [file Data_Sheet_1.pdf]

(A)

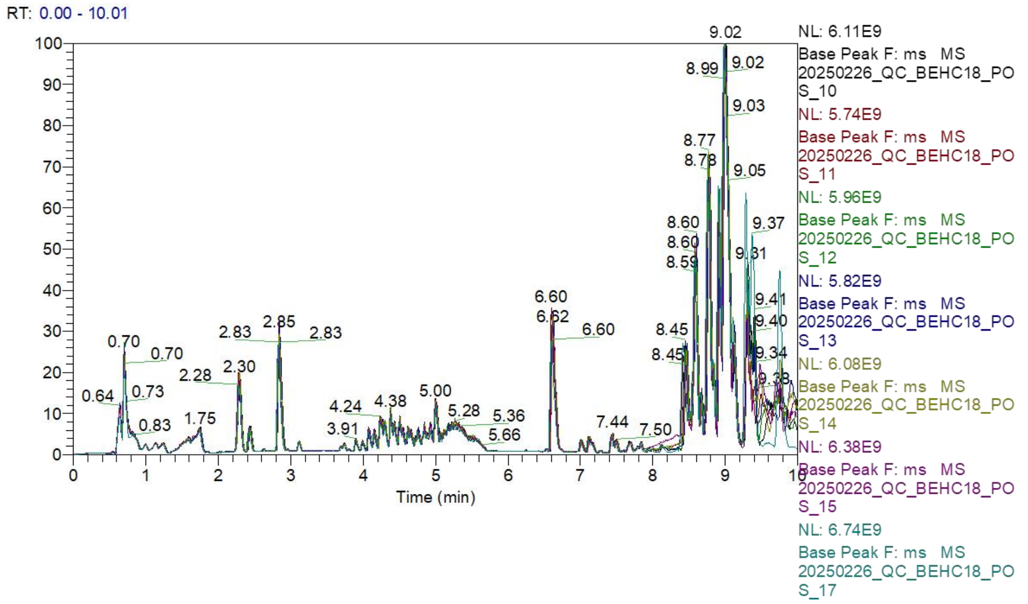


(B)

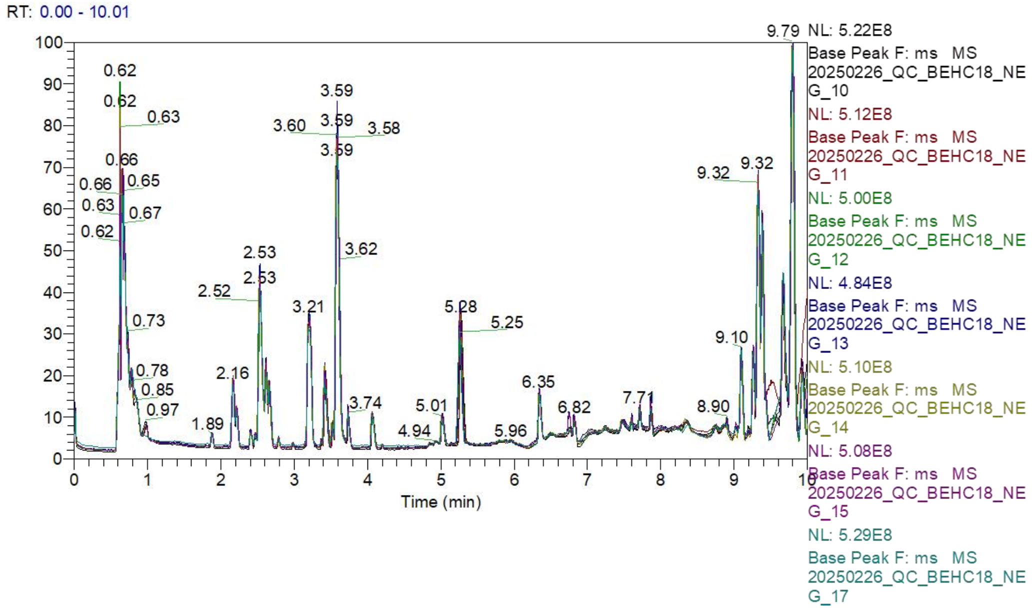


**Figure 1.** QC of UPLC-MS Analysis. (A)Positive mode ;(B)Negative ion mode.

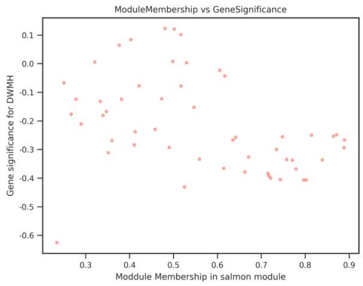
ModuleMembership vs GeneSignificance
Gene significance for DWMH
Moddule Membership in salmon module

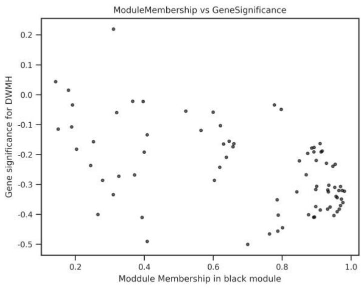
ModuleMembership vs GeneSignificance
Gene significance for DWMH
Moddule Membership in black module

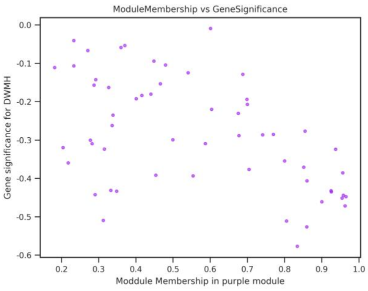
ModuleMembership vs GeneSignificance
Gene significance for DWMH
Moddule Membership in purple module

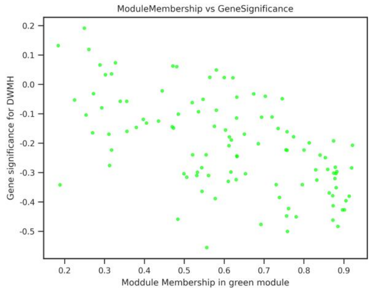
ModuleMembership vs GeneSignificance
Gene significance for DWMH
Moddule Membership in green module

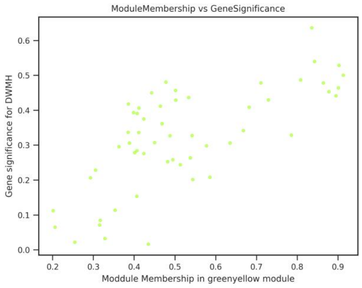
ModuleMembership vs GeneSignificance
Gene significance for DWMH
Moddule Membership in greenyellow module

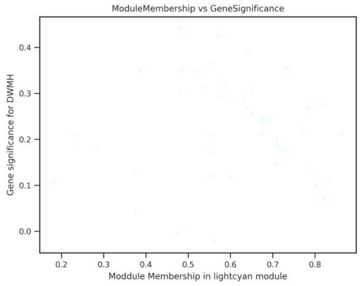
ModuleMembership vs GeneSignificance
Gene significance for DWMH
Moddule Membership in lightcyan module

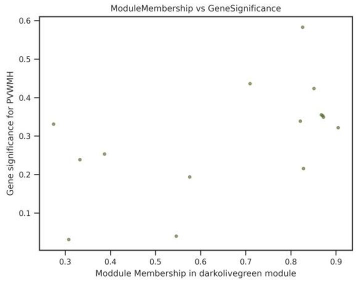
ModuleMembership vs GeneSignificance
Gene significance for PVWMH
Moddule Membership in darkolivegreen module

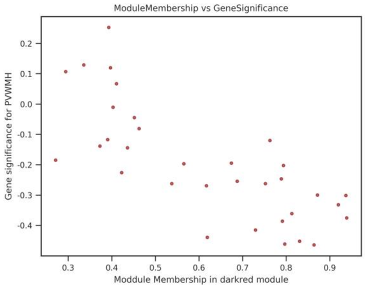
ModuleMembership vs GeneSignificance
Gene significance for PVWMH
Moddule Membership in darkred module

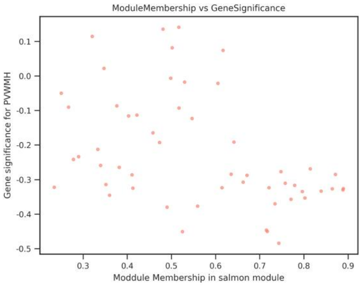
ModuleMembership vs GeneSignificance
Gene significance for PVWMH
Moddule Membership in salmon module

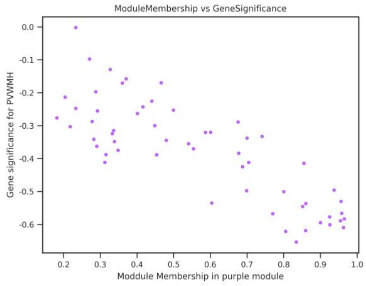
ModuleMembership vs GeneSignificance
Gene significance for PVWMH
Moddule Membership in purple module

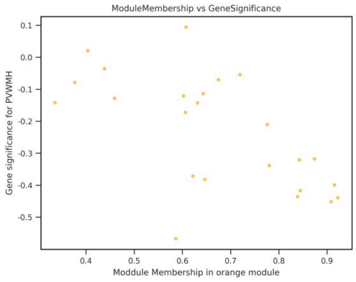
ModuleMembership vs GeneSignificance
Gene significance for PVWMH
Moddule Membership in orange module

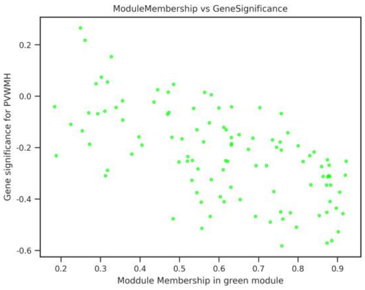
ModuleMembership vs GeneSignificance
Gene significance for PVWMH
Moddule Membership in green module

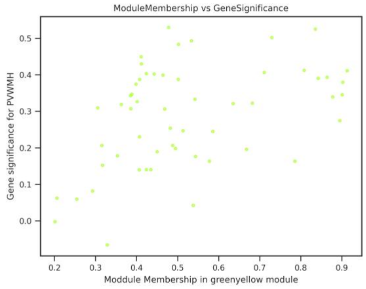
ModuleMembership vs GeneSignificance
Gene significance for PVWMH
Moddule Membership in greenyellow module

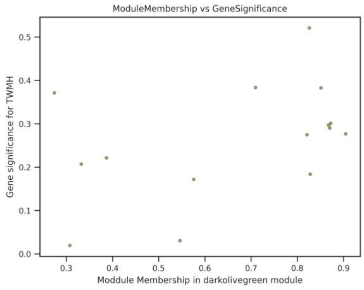
ModuleMembership vs GeneSignificance
Gene significance for TWMH
Moddule Membership in darkolivegreen module

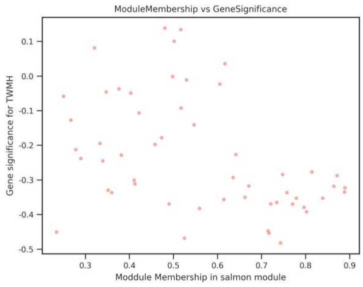
ModuleMembership vs GeneSignificance
Gene significance for TWMH
Moddule Membership in salmon module

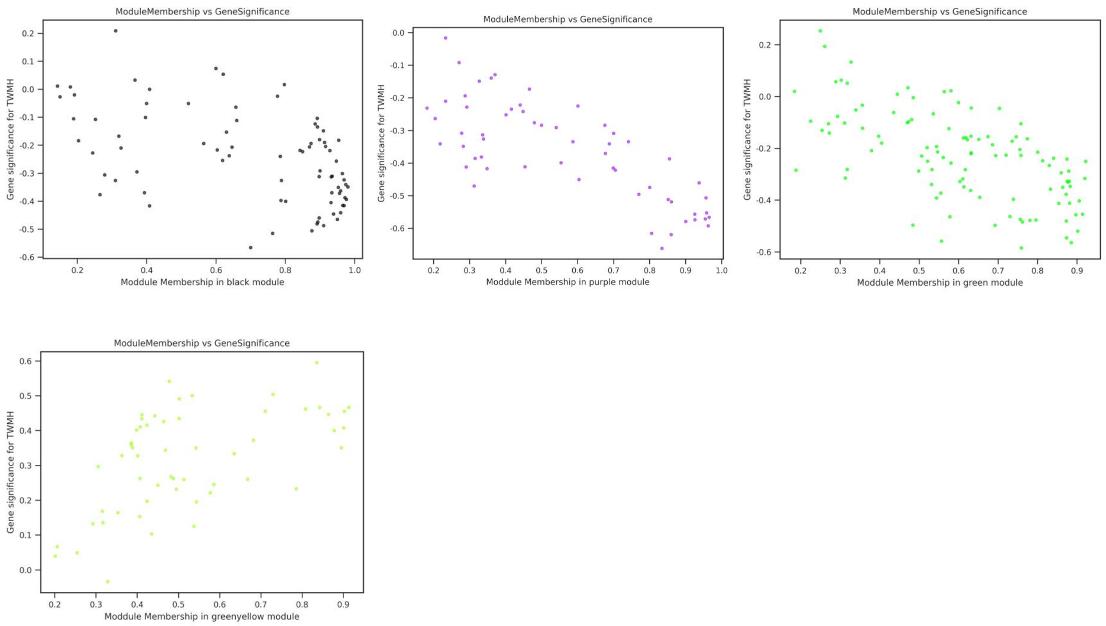


**Supplementary Figure 2** Correlation between module membership and gene significance of all metabolites in DWMH, PVWMH and TWMH.
